# Bacterial Protein Homeostasis Disruption as a Therapeutic Intervention

**DOI:** 10.3389/fmolb.2021.681855

**Published:** 2021-06-02

**Authors:** Laleh Khodaparast, Guiqin Wu, Ladan Khodaparast, Béla Z. Schmidt, Frederic Rousseau, Joost Schymkowitz

**Affiliations:** ^1^ Switch Laboratory, VIB Center for Brain and Disease Research, Leuven, Belgium; ^2^ Switch Laboratory, Department of Cellular and Molecular Medicine, Leuven, Belgium

**Keywords:** protein homeostasis, protein aggregation, antibacterial peptide, Pept-in, inclusion body, aggregation-prone region, adsorption, advanced oxidation processes

## Abstract

Cells have evolved a complex molecular network, collectively called the protein homeostasis (proteostasis) network, to produce and maintain proteins in the appropriate conformation, concentration and subcellular localization. Loss of proteostasis leads to a reduction in cell viability, which occurs to some degree during healthy ageing, but is also the root cause of a group of diverse human pathologies. The accumulation of proteins in aberrant conformations and their aggregation into specific beta-rich assemblies are particularly detrimental to cell viability and challenging to the protein homeostasis network. This is especially true for bacteria; it can be argued that the need to adapt to their changing environments and their high protein turnover rates render bacteria particularly vulnerable to the disruption of protein homeostasis in general, as well as protein misfolding and aggregation. Targeting bacterial proteostasis could therefore be an attractive strategy for the development of novel antibacterial therapeutics. This review highlights advances with an antibacterial strategy that is based on deliberately inducing aggregation of target proteins in bacterial cells aiming to induce a lethal collapse of protein homeostasis. The approach exploits the intrinsic aggregation propensity of regions residing in the hydrophobic core regions of the polypeptide sequence of proteins, which are genetically conserved because of their essential role in protein folding and stability. Moreover, the molecules were designed to target multiple proteins, to slow down the build-up of resistance. Although more research is required, results thus far allow the hope that this strategy may one day contribute to the arsenal to combat multidrug-resistant bacterial infections.

## Targeting Multiple Targets Yields More Robust Antibacterials

Most currently used antibacterial approaches target an essential protein or process (either directly or indirectly) in one of these four categories: nucleic acids synthesis, proteins synthesis, the synthesis or integrity of the bacterial cell wall or bacterial membrane, and folic acid metabolism (Kapoor et al., 2017). Antibiotics targeting one single protein have been favored in the past because these single-target antibiotics can offer high target specificity and induce fewer side effects. Having a single target, however, sets up the rapid generation of resistance since only one protein or pathway needs to be circumvented to develop resistance to the antibiotic. Combination therapy has been a useful approach to overcome bacterial resistance but it works even better by combining multi-target antibiotics (Oldfield and Feng, 2014). Resistance has been observed on average 2 years after marketing an antibiotic (Clatworthy et al., 2007; Coates et al., 2011) and the experience has been that target-related spontaneous resistance develops more rapidly if the antibiotic has a single target than if the antibiotic affects several targets in parallel and/or those targets are encoded by multiple genes (Brötz-Oesterhelt and Brunner, 2008; Gray and Wenzel, 2020). In general, a single mutation in the target may be sufficient to develop high-level target-related resistance against antibiotics that have a single target encoded by one gene. At the same time, multiple mutations or acquired resistance genes are required to evolve a substantial level of resistance against antibiotics that affect several targets in parallel and/or if the targets are encoded by multiple genes. Vancomycin is a good example of an antibiotic that requires the acquisition of multiple genes for developing resistance. Vancomycin compromises cell envelope integrity (Stogios and Savchenko, 2020) by binding to the D-Ala-D-Ala moiety of un-crosslinked lipid II and inhibiting autolytic enzymes by binding to free C-terminal D-Ala-D-Ala residues in the mature cell wall (Sieradzki and Tomasz, 2006). Although modifying lipid II to D-Ala-D-lac or D-Ala-D-Ser can render bacteria vancomycin-resistant, it is rather difficult to achieve these modifications. Indeed, bacteria that achieve a high-level resistance to vancomycin do so by expressing several (five or more) newly acquired genes (Okano et al., 2017; Stogios and Savchenko, 2020). Due to the difficulty of developing resistance against vancomycin, the first discovery of resistant strains occurred almost 30 years after its initial clinical use.

One strategy to create multi-target antibiotics has been modifying existing antibiotics to increase the number of targets and/or pathways affected, which subsequently overcomes the existing resistance mechanisms and delays the occurrence of novel resistance. For example, the second-generation macrolide azithromycin exerts a more potent antimicrobial activity by inducing membrane permeability in addition to inhibiting protein synthesis (Gh et al., 2018). In the case of vancomycin, target range broadening was achieved with oritavancin, a derivative that not only binds D-Ala-D-Ala containing lipid II but also D-Ala-D-lac lipid II precursors, thereby addressing one of the resistance mechanisms to vancomycin. (Stogios and Savchenko, 2020). In addition, it also inhibits transpeptidation and may affect RNA synthesis, as well (Zeng et al., 2016). Although we cannot predict if oritavancin will have such a long career as vancomycin, resistance to it has not been reported yet.

Other antibiotics affecting multiple targets/biological pathways through a novel mode of action have also been developed. Recent progress in this field includes teixobactin (Ling et al., 2015), SCH-79797 (Martin et al., 2020), corbomycin and complestatin (Culp et al., 2020). Discovered in a screen of uncultured bacteria, teixobactin seems to have evolved to minimize resistance development by target microorganisms (Ling et al., 2015). This novel antibiotic inhibits bacterial cell wall synthesis by capturing precursors such as Lipid I, Lipid II, Lipid III, and undecaprenyl pyrophosphate (Shukla et al., 2020) and its ability to interfere with multiple targets is probably why resistance to it could not be detected (Ling et al., 2015). Similarly, bacteria showed no sign of resistance to the recently described SCH-79797 after passaging them for 30 days at a concentration of SCH-79797 that is lower than its minimal inhibitory concentration (MIC) (Martin et al., 2020). SCH-79797 is bactericidal toward both Gram-negative and Gram-positive bacteria by disrupting folate metabolism and the integrity of the bacterial membrane (Martin et al., 2020). Corbomycin and complestatin bind and subsequently block the function of a broad range of structurally unrelated autolysins, thereby inhibiting peptidoglycan remodeling of the cell wall during growth (Culp et al., 2020). Corbomycin was also shown to be able to inhibit fatty acid synthesis (Kwon et al., 2015). Although a low level of resistance was reported for corbomycin and complestatin (resistant mutants have mutations in autolysin proteins), single-gene deletions changed susceptibility only 2-fold or less (Culp et al., 2020).

Although multi-targeted antibiotics are not immune to inactivating mechanisms that either block their uptake, increase their efflux or promote their degradation, the studies above suggest that the chance of a target-based high-level endogenous resistance is lower for multi-target antibiotics, which explains why they have been gaining increasingly more attention (Tyers and Wright, 2019; Gray and Wenzel, 2020). The case of vancomycin also showed that it is not only the number of targets that matters but also the difficulty to modify that target. Therefore, the optimal antibiotic strategy has multiple targets, each of which is hard to be genetically deleted or altered by random mutations under selective pressure. In what follows, we explore the idea that perturbation of the protein homeostasis network via inducing aggregation of bacterial proteins could constitute such an attractive antibiotic strategy.

## Bacterial Proteostasis Faces Particular Challenges

Protein homeostasis, also called proteostasis, is a term used to describe all protein quality control activities of the (eukaryotic or prokaryotic) cell including protein synthesis, folding, translocation and degradation. Given that as good as all biological activity in a cell is mediated by proteins, proteostasis is a fundamental component of cellular life, consuming about half of the metabolic energy (Buttgereit and Brand, 1995). The cells have evolved a complex and interconnected quality control system, called the proteostasis network (PN), to support the integrity and functionality of the proteome under physiological conditions and to protect the proteome against acute stress conditions. The PN consists of chaperones, proteases as well as other specialized molecules (Mogk et al., 2011; Kampinga et al., 2019). The importance of proteostasis for the health of the organism (Balch et al., 2008) and the decline of proteostasis during ageing (Ben-Zvi et al., 2009) have been recognized for over a decade.

Even though the general principles of protein folding are similar in all organisms, maintaining proteostasis is especially challenging for bacteria due to their small volume, the lack of membrane-separated compartments, and high protein turnover rates. Additionally, bacteria are constantly subject to stress conditions, including heat/cold shock, oxidative stress, osmotic shock, heavy metal toxicity, changes in hydrostatic pressure, the presence of drugs, as well as host organism mounted-stresses in response to infections such as chemical stresses (e.g. reactive oxygen and nitrogen species), the presence of antibiotics or the elevated temperature from fever (Ehrt and Schnappinger, 2009; Dahl et al., 2015; Harnagel et al., 2020). The exposure of bacteria to these pressures as well as the complexity of metabolic changes that arise in response to these pressures can cause significant perturbations of bacterial proteostasis (Morano et al., 2012; Gayán et al., 2017). Depletion of intracellular ATP can also drive protein aggregation because maintaining proteostasis consumes a lot of energy and ATP is a biological hydrotrope that helps to keep hydrophobic proteins in solution (Patel et al., 2017; Pu et al., 2019).

Both the short doubling times of bacteria (*E. coli* doubling time is about 20 min) and adaptation to changing conditions require a high protein turnover rate, and indeed the speed of protein translation is at least five times faster in bacteria than in eukaryotes (de Groot and Ventura, 2010). In a recent study (Ramakrishnan et al., 2019), we have shown that protein abundance and translation speed are strong determinants of chaperone-dependence in *E. coli* and by extension, likely other bacterial strains and species, as well. So, although certain complex folded proteins intrinsically need assistance from chaperones likes GroEL to fold, most fast-translated proteins require the help of trigger factor and DnaK, regardless of whether they are intrinsically capable of independent folding. Upon the genetic deletion of these factors, proteins tend to end up in the insoluble fraction, likely undergoing aggregation (Deuerling et al., 2003; Chapman et al., 2006; Hartl et al., 2011).

A higher protein turnover rate implies more individual polypeptide chains are in the course of translation or folding at any given time. Since the chance of aggregation is the highest during translation before the protein gains its native structure (Willmund et al., 2013), it is, therefore, likely that a higher protein turnover rate renders the proteostasis of bacteria more vulnerable to perturbations (Beerten et al., 2012). The idea of targeting the proteostasis of quickly dividing cells is also being exploited in human cells in the forms of promising cancer treatments based on pharmacologic inhibition of, for example, Hsp70 or Hsp90 (Hipp et al., 2014), where the difference in translation rate is one element that helps create a therapeutic window between cancer cells and their healthy counterparts.

However, in apparent contradiction with these ideas, bacteria show remarkable resilience to aggregation, notably in the expression of heterologous proteins, some of which end up in massive inclusions bodies consisting of aggregated forms of the protein and occupying a significant fraction of the cellular volume. Although the production of such a recombinant protein may impart such a metabolic burden on the microorganism that can cause a considerable delay in generation time (Rosano and Ceccarelli, 2014), it is often not lethal. This suggests that the aggregation of a heterologous protein is contained and does not lead to a proteostatic collapse.

To what extent inclusion body formation upon heterologous expression can be related to protein translation rates is unclear since many factors such as post-translational modifications and co-evolution with chaperones may also play a role. But it could be argued that as proteins got larger and more complex during evolution (Netzer and Hartl, 1997; Balchin et al., 2016), translation speed had to be reduced to give proteins more time for co-translational folding and to prevent aggregation. This seems to make perfect sense since expressing eukaryotic proteins in bacteria at a slower speed reduces their aggregation (Siller et al., 2010) and many experiments show that a higher translation elongation speed results in more aggregation both in bacteria and in eukaryotes. E.g., speeding up the translation of the cystic fibrosis transmembrane conductance regulator in eukaryotic cells resulted in a higher amount of aggregated protein (Kim et al., 2015), and our lab has shown in bacteria that increasing the translation rate of a transcript resulted in more insoluble protein (Ramakrishnan et al., 2019). Cooling down the cultures often resolves aggregation of heterologously expressed proteins (Rosano and Ceccarelli, 2014). This also seems to support the apparent detrimental effect of high translation speed on protein folding, since culturing bacteria at a lower temperature would certainly provide an overall reduction in translation rates (although it may have many other effects, as well).

However, most experiments increase the translation speed of a transcript by codon optimization, i.e. eliminating rare codons by replacing each codon with a faster-translating counterpart. Codon-optimization not only speeds up translation but can also perturb the rhythm of translation by eliminating the pauses associated with rare codons. As it has become clear recently, the rate of elongation is not uniform along the mRNA and one of the factors influencing elongation speed is codon usage (Liu, 2020; Samatova et al., 2021). Rare codons are translated somewhat slower and an increasing number of studies suggests that co-translational folding is a sequential event in which the presence of rare codons establishes transcriptional pauses that provide enough time for the nascent protein to acquire the correct conformation (Sabate et al., 2010). Moreover, although the high speed of bacterial translation makes folding difficult for eukaryotic proteins, probably due to their multi-domain structure (Netzer and Hartl, 1997), slowing down or speeding up translation seems to make no difference for bacterial proteins (Siller et al., 2010). Therefore, it is changing the rhythm of translation that increases misfolding and aggregation and not the higher speed of translation (Liu, 2020; Samatova et al., 2021). It seems that the high volume of protein turnover makes the proteostasis of bacteria vulnerable and not the high speed of translation itself.

## Bacterial Proteostasis as a Target for Antimicrobials

The proteostasis network (PN) maintains cellular proteins in a state that allows optimum biological activity while responding to environmental stimuli, starting with the synthesis of new polypeptide chains, through the folding of newly translated proteins to the repair, disaggregation or degradation of damaged proteins that unfold or aggregate, in particular under stress conditions (Powers and Balch, 2013). Balch *et al.* proposed the downregulation of bacterial proteostasis as an antibacterial strategy in 2008 (Balch et al., 2008) but antibiotics that tamper with proteostasis by targeting one of the principal components of the PN, the ribosome, have been around for much longer. Aminoglycosides, tetracyclines, macrolides, lincosamides, etc. interfere with protein synthesis and cause a proteostasis imbalance by disrupting translational fidelity, causing premature termination of translation, preventing the binding of t-RNAs to the ribosome or causing the premature detachment of incomplete peptide chains from it (Ling et al., 2012; Kapoor et al., 2017).

Many examples show that causing bacterial chaperone deficiency may also be an effective way to limit bacterial viability or can reduce antibiotic tolerance of pathogenic species (Lee et al., 2016). Genetic deletion of chaperones involved in protein folding, like GroEL, trigger factor or DnaK, causes “an avalanche” of aggregation (Deuerling et al., 2003; Chapman et al., 2006) that poses a heavy burden on the bacteria and limits their resistance to stresses. The redundancy of chaperones gives bacteria some resiliency against such attacks, though. For example, DnaK/DnaJ and TF have overlapping sets of substrates and one can compensate for the absence of the other—but a combined deletion of both is lethal above 30°C (Deuerling et al., 1999; Deuerling et al., 2003). Similarly, while the individual loss of neither HtpG (an Hsp90-homologue) nor ClpB (a disaggregase) is lethal to *Mycobacterium tuberculosis*, cells lacking both these chaperones become hypersensitive to host-like stresses and go into a nonreplicating state (Harnagel et al., 2020). Tampering with the clearance of protein aggregates also has severe consequences for bacteria. For example, cells lacking the ClpB disaggregase become more sensitive to heat or oxidative stress (Harnagel et al., 2020). Based on these observations, inhibitors targeting the chaperone system such as DnaK inhibitors (Czihal et al., 2012) and HSP60/10 chaperonin system inhibitors (Stevens et al., 2019) have been put forth as antibiotic strategies but it remains to be seen whether sufficient specificity toward bacterial chaperones over mammalian counterparts can be achieved.

It has also become apparent in recent years that antimicrobial peptides (AMPs) that were initially considered only as agents that disrupt bacterial membranes, also interact with intracellular targets, including PN components (Nguyen et al., 2011; Lee and Lee, 2015; Lazzaro et al., 2020). For example, the primary target of the proline-rich AMP oncocin is thought to be the ribosome exit channel (Roy et al., 2015; Seefeldt et al., 2015). Oncocin also binds to and inhibits the bacterial Hsp70 homolog DnaK (Knappe et al., 2011), one of the key chaperones in bacteria, which will likely amplify the disruption of bacterial proteostasis by this peptide. Interestingly, many AMPs form amyloid structures spontaneously (Zhao et al., 2006; Mahalka and Kinnunen, 2009; Torrent et al., 2011) and some AMPs co-aggregate with bacterial proteins (Code et al., 2009).

All these targeted approaches that specifically interfere with various components of the PN and meddle with the synthesis or folding of proteins, or the clearance of protein aggregates have a common feature: they all produce a large pool of aggregated proteins. The accumulation of damaged, misfolded or aggregated proteins as a sign of the decline of proteostasis has been studied extensively in eukaryotes where it contributes to ageing and senescence (Taylor and Dillin, 2011; Santra et al., 2019). Although it is controversial whether bacteria undergo ageing due to the accumulation of aggregated proteins (Rosano and Ceccarelli, 2014; Schramm et al., 2019), the accumulation of protein aggregates can affect the growth rate, stress resistance and virulence of bacteria, as well (Schramm et al., 2019). Protein aggregation appears to play a role in causing bacterial death in certain lethal conditions such as heat and exposure to heavy metals, either through massive protein aggregation leading to proteostasis collapse or the depletion of certain essential factors (Ling et al., 2012; Tamás et al., 2014; Bednarska et al., 2016; Khodaparast et al., 2018; Katikaridis et al., 2019). On the flip side, the importance of a highly competent proteostasis machinery for bacterial virulence is underlined by the fact that a transmissible locus for protein quality control (TLPQC-1) spreads by horizontal gene transfer amongst pathogenic strains (Lee et al., 2016), apparently conferring fitness benefits to the pathogens during infection.

It seems, therefore, that despite the stress-adaptive transcriptional programs bacteria can initiate to deal with proteostasis imbalance (Schramm et al., 2019), targeting bacterial proteostasis can indeed be an effective antibacterial strategy, either as a standalone treatment or in conjunction with existing antibiotics. Although the PN can increase its capacity dramatically on-demand, it is possible to overwhelm the cellular machinery that deals with damaged proteins, leading to and causing proteostatic collapse. As we saw, such perturbation of the bacterial proteostasis can be achieved either by interfering with one or more specific components of the PN or by creating such a large pool of aggregated proteins within the cell that its clearance exceeds the capacity of the PN. In the next sections, we will see how this latter can be achieved.

## Amyloid-like Aggregation can be Seeded in a Sequence-Specific Manner

**FIGURE 1:**
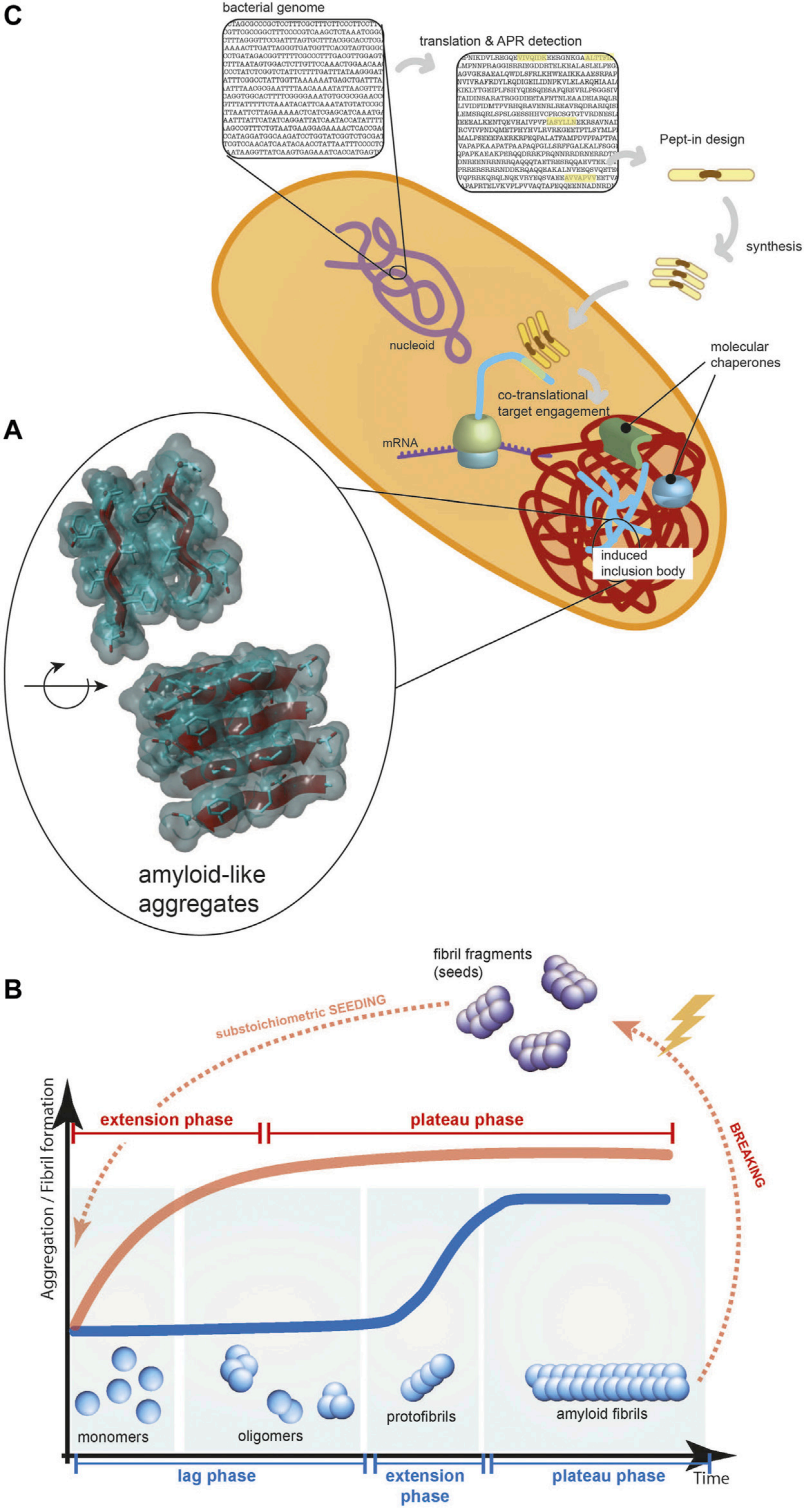
**(A)** The core of protein aggregates contains beta-strands forming beta-sheets held together by hydrogen bonds between the polypeptide backbones and these beta-sheets can pack in layers via interdigitating amino acid sidechains. **(B)** The typical kinetics of amyloid-like protein aggregation (blue line): a rate-limiting nucleation phase, a fast-growing elongation phase, and a final plateau phase. Seeding the reaction with substoichiometric amount of fibril fragments can eliminate the lag phase (red line). **(C)** Pept-ins are designed based on detecting APRs in the translated bacterial genome. They seem to form small seeds (depicted as stacks of Pept-ins in the Figure) that induce the fast co-translational aggregation of proteins and the formation of inclusion bodies.

Protein aggregation has been (and by some perhaps still is) considered to be a non-specific process: a phase separation driven by clusters of hydrophobic residues in misfolded proteins. Our increasing structural understanding of protein aggregates over the last two decades has demonstrated that, both *in vitro* and *in vivo*, protein aggregates are much more structured macromolecular assemblies (Morell et al., 2008) than previously thought. The most predominant mechanism of aggregation is amyloid-like aggregation, which is based on the interactions of beta-strands from different polypeptides forming intermolecular beta-sheets (Figure 1A). Intracellularly, protein aggregates often accumulate into a range of inclusions, the specifics of which differ between organisms and cell types, but the aggregates they contain have been shown to share the basic beta-sheet-rich structure.

In bacteria, the term inclusion body (IBs) is widely used to indicate such aggregate-rich structures, formed, e.g. when the bacterial cellular machinery is unable to fold an over-expressed protein in its native conformation. It is now clear that these are “not mere amorphous graveyards” (Otzen, 2010) but have amyloid-like properties including high beta-sheet content (Carrió et al., 2005; Ventura and Villaverde, 2006; Garcia-Fruitos et al., 2011; Upadhyay et al., 2012; Khodaparast et al., 2018). The structure of the most highly ordered protein aggregates, amyloid fibres (Sawaya et al., 2007), has been investigated in detail and showed that both amyloid aggregates formed *in vitro* or those extracted *ex-vivo* have a generic cross-beta backbone organization as revealed by X-ray diffraction data (Serpell et al., 1995; Sunde et al., 1997; Nelson et al., 2005; Sawaya et al., 2007) or more recently by reconstruction of cryo-electron microscopy images of full-length fibrils (Lu et al., 2013; Fitzpatrick et al., 2017; Gremer et al., 2017; Falcon et al., 2018; Falcon et al., 2019).

In the core of amyloid fibers, identical sequences in a beta-strand conformation are stapled together into beta-sheets through hydrogen bonds between the polypeptide backbones, as well as the lateral stacking of the side chains of consecutive strands, and these beta-sheets can further pack laterally via tightly interdigitated sidechains forming stable structures, known as “*steric zippers*” (Figure 1A) (Nelson et al., 2005; Sawaya et al., 2007; Rodriguez et al., 2015). An interesting recent exploitation of the similarity between the structure of bacterial IBs and disease-causing amyloids is using bacteria to screen for anti-amyloid (beta-blocker) drugs for conformational diseases (Caballero et al., 2019).

Amyloid-like protein aggregation follows a typical sigmoidal curve, initiated with a rate-limiting nucleation phase, followed by a fast-growing elongation phase and ending with a final plateau phase (Figure 1B) (Knowles et al., 2009; Arosio et al., 2016; Lutter et al., 2019). Although the amyloid aggregate state of many proteins is thermodynamically more stable than the soluble form under conditions found *in vivo*, there is a kinetic barrier towards amyloid formation, partly because the conformational freedom of the peptide backbone contributes to the entropy of the system (Buell et al., 2014). During the slow and thermodynamically unfavorable nucleation phase, stable seeds are formed by rearranging misfolded protein structures into a series of beta-strands.

When the concentration of seeds is high enough, the growth of seeds becomes the dominant process and protein aggregation proceeds to the elongation or extension phase (Figure 1B). This is the fastest phase of the overall aggregation reaction, *by several orders of magnitude* (Buell, 2019). In this phase, the fibrils grow in a direction parallel to the fibril axis by adding monomeric building blocks to the fibril end, during which the protein monomers adopt the cross-beta structure of the seeds as a template (Soto and Pritzkow, 2018; Lutter et al., 2019). In this phase, new seeds are continually formed through fragmentation of the growing aggregates and secondary nucleation, i.e. the formation of new seeds on the surface of the aggregates, which appear to act as catalysts. The most important intrinsic barrier to protein aggregation can be circumvented by supplying pre-formed seeds to a sample of fresh monomer (Figure 1B) and this has been shown to work both in a test tube (O’Nuallain et al., 2004; Saijo et al., 2017), in cells (Colby et al., 2007; Holmes et al., 2014) and in mouse models *in vivo* (Hamaguchi et al., 2012; Falcon et al., 2015; Narasimhan et al., 2017; Gomes et al., 2019).

There is a controversy over whether IB formation in bacteria is an active, protective cellular process that deposits aggregates as IBs at specific polar region(s) or IB formation depends only on the physical interaction of the protein chains moving around purely by Brownian motion and IBs end up at the cell pole because they are crowded out from the middle of the cell by nucleic acids (Tyedmers et al., 2010; Coquel et al., 2013; Rinas et al., 2017). Whichever the case may be, IBs appear to be “built” in a selective way and at least some of this selectivity can be contributed by a diffusion-driven (not active) mechanism driven by the polypeptide chains themselves. As it has been demonstrated *in vitro* with many proteins, the polypeptide chains themselves can produce aggregates of a homogeneous composition (O’Nuallain et al., 2004; O’Nuallain et al., 2005; Wetzel, 2006), and co-expression experiments also showed that non-homologous aggregation-prone proteins initially deposit in separate inclusion bodies both in bacteria (Morell et al., 2008) and eukaryotic cells (Rajan et al., 2001). IBs contain predominantly the over-expressed protein and their properties depend on the protein being over-expressed (Upadhyay et al., 2012)—although they do engulf other bystanders like small heat-shock proteins IbpA and IbpB and the main chaperones DnaK and GroEL (Ventura and Villaverde, 2006), which may also be part of the machinery to build a well-ordered IB.

The tight packing of side chains at the core of amyloid fibrils suggests that amyloid aggregates are not only structured but the assembly of such structures is also selective and even sequence-specific (O’Nuallain et al., 2004). The sequence specificity of amyloid aggregation has been demonstrated using seeding experiments, as well. *In vitro* seeding experiments suggest that seeding between identical sequences is favored (O’Nuallain et al., 2004; O’Nuallain et al., 2005; Wetzel, 2006), although there are examples of cross-seeding between similar but non-homologous sequences, e.g. cross-seeding between amyloid beta peptide (Abeta) and Islet Amyloid Polypeptide (IAPP, also called amylin) (Oskarsson et al., 2015) or the Abeta peptide and alpha-synuclein (Ono et al., 2012) or lysozyme and other proteins (Krebs et al., 2004).

## Short Polypeptide Segments Control Aggregation

The selectivity of protein aggregation and the tightly packed structure of amyloid aggregates suggest that certain sequence fragments within a polypeptide chain would be more suitable to incorporate in such structures than others. Many groups have developed bioinformatics algorithms to detect regions, called aggregation-prone regions (APRs), in polypeptide sequences that would be particularly suitable for forming aggregates (Conchillo-Sole et al., 2007; Tsolis et al., 2013; Walsh et al., 2014; Espargaró et al., 2015). Our laboratory has contributed with TANGO (Fernandez-Escamilla et al., 2004), WALTZ (Maurer-Stroh et al., 2010) and more recently Cordax (Louros et al., 2020).

We have used our aggregation prediction algorithms to show that APRs are present in almost any protein in any given proteome, whether prokaryotic or eukaryotic (<5% of protein domains have no APRs) (Rousseau et al., 2006; Ganesan et al., 2016). These findings have been confirmed by other labs using different prediction algorithms (Monsellier et al., 2008; Goldschmidt et al., 2010; Rawat et al., 2018). APRs are generally short (5–15 residues long) sequences that have an intrinsic propensity to self-associate by beta-strand interactions (Rousseau et al., 2006; Goldschmidt et al., 2010) and their role in inducing protein aggregation has been confirmed experimentally. We know that the presence of APR(s) in a polypeptide chain is both necessary and sufficient for inducing protein aggregation. APRs are necessary for protein aggregation because introducing point-mutations that abolish the aggregation propensity of an APR reduce the aggregation propensity of the entire protein (Ganesan et al., 2016). And APRs are sufficient for inducing protein aggregation because grafting APRs of known amyloid-associated proteins onto proteins that do not aggregate by themselves render them aggregation-prone (Ventura et al., 2004; Teng and Eisenberg, 2009). The mentioned cryo-EM structures of amyloid fibrils extracted from patients show the involvement of a much larger segment of the polypeptide chain in the final amyloid fibril structure than just the APRs (Lu et al., 2013; Fitzpatrick et al., 2017; Gremer et al., 2017; Falcon et al., 2018; Falcon et al., 2019), but the APRs are still the focal points for initiating aggregation. The beta-strands formed by the APRs are part of the beta-sheets in the fibril core and they form the “aggregation hot spots” that kinetically control amyloid formation while the regions flanking APRs can either promote or inhibit aggregation and modulate the structure of the fibers (Sumner Makin and Serpell, 2004; Savastano et al., 2020; Ulamec et al., 2020; Zhang et al., 2020).

Most proteins possess at least one APR, and they usually form either part of the hydrophobic core of globular proteins or interaction sites that become buried in e.g. through protein-protein interactions. The few solvent-exposed APRs in native proteins are generally APRs contributing to protein interaction interfaces or catalytic sites (Ventura et al., 2002; Prabakaran et al., 2017). Since most APRs are buried, they represent a danger for aggregation only in situations where proteins are partially or completely unfolded, such as during protein translation or translocation, under situations of physiological stress or due to mutations that destabilize the native conformation (Ganesan et al., 2016; Langenberg et al., 2020). We have shown that APRs are not just located in the hydrophobic core of proteins, there is a deep entanglement between protein stability and protein aggregation propensity that means that aggregation propensity is as evolutionarily conserved as the structure itself (Langenberg et al., 2020). As a consequence, APRs constitute interesting targets for the development of antibiotics since these regions are the least likely to accumulate mutations in the short term.

## Targeted Protein Aggregation

The aggregation of a wide range of proteins has been described to follow the classic sigmoidal aggregation kinetics in many organisms, including bacteria, fungi and mammals, forming either pathogenic or functional amyloids (Platt et al., 2008; Seuring et al., 2012; Van Gerven et al., 2015; Villar-Pique et al., 2016), meaning that aggregation is controlled at the stage of seed formation and then speeds up once enough seeds are available.

**FIGURE 2:**
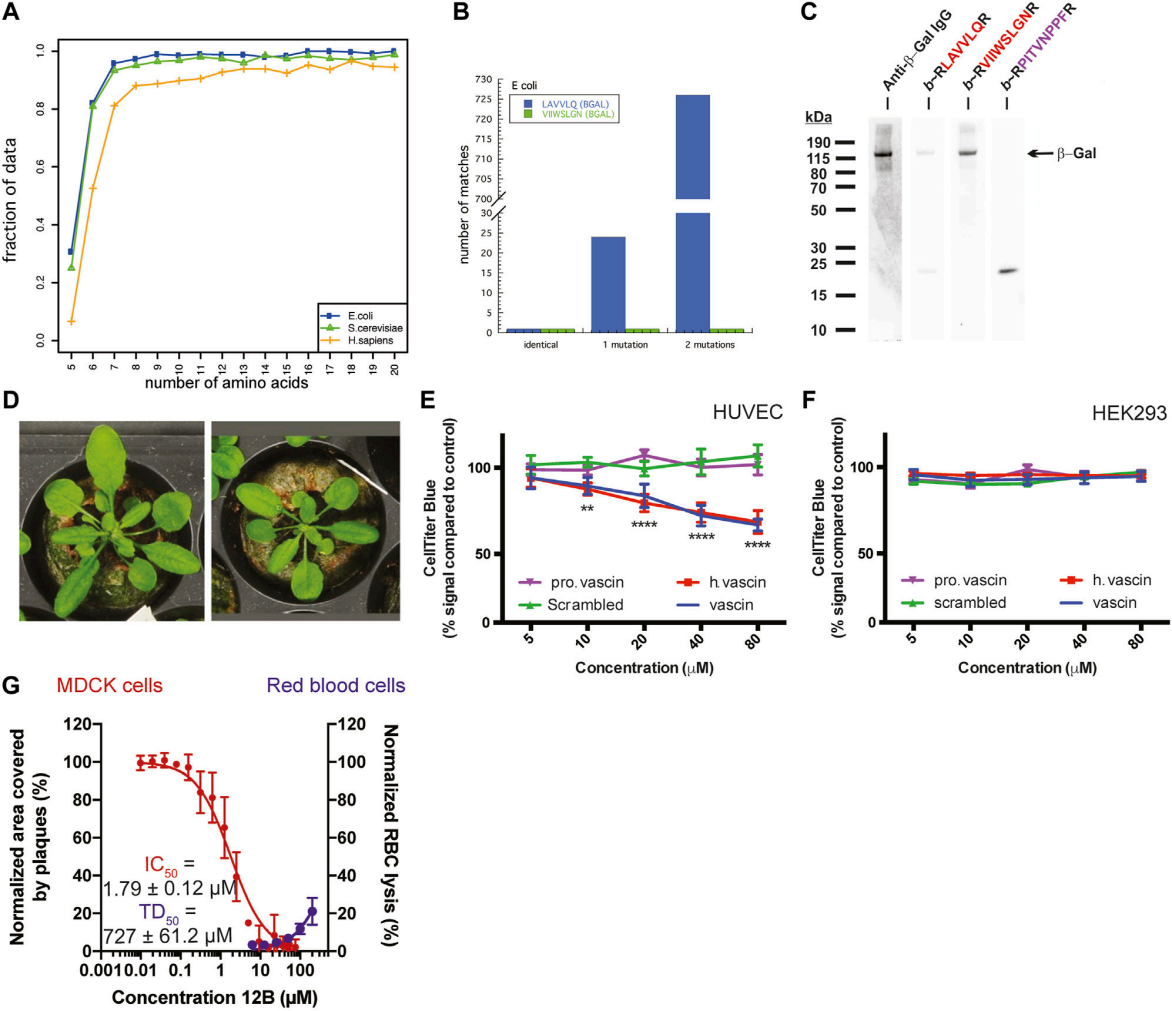
**(A)** The fraction of APRs that are unique within the *E. coli*, *S. cerevisiae*, or *H. sapiens* proteome, plotted by length of the APR. **(B)** The number of sequences in the *E. coli* proteome that match two peptides derived from β-galactosidase (allowing 0, 1, or 2 substitutions). **(C)** Detection of β-Galactosidase in bacterial cell lysates with immunoblotting using specific antibody (lane 1) or with PepBlot using sequence-specific peptides (lanes 2–4) (**A–C** adapted from Ganesan et al., 2015). **(D)** Arabidopsis plants expressing a Pept-In targeting the negative regulator of brassinosteroid signaling **(left)** grow larger than wild type plants **(right)**. Adapted from Betti et al., 2016. **(E,F)** Dose-dependent toxicity of vascin, its human counterpart (h vascin), and a proline mutant of vascin (pro vascin) or scrambled version as controls (from 2.5 to 100 mm) by the CellTiter-Blue assay. **(E)** Vascin and h.vascin are toxic to HUVEC cells that depend on VEGFR signaling for survival but not to HEK293 cells **(F)** (**E,F** adapted from Gallardo et al., 2016). **(G)** Dose-dependent effect of an antiviral peptide (12B) targeting an APR in the cap-binding domain of polymerase basic protein 2 of the influenza A virus. Treating MDCK cells infected with influenza A led to a dose-dependent decrease of the area covered by viral plaques (red curve, left axis) with an IC50 below 2 μm. Data are normalized to buffer-treated cells and the mean ± SD of 4 independent experiments is shown. Peptide 12B did not have significant hemolytic activity (blue curve, right axis). For toxic dose (TD50): data are normalized to buffer-treated (0% lysis) and 0.1% Triton-treated cells (100% lysis) and the mean ±SD of 3 independent experiments is shown. (**G** Adapted from Michiels et al., 2020).

Analyzing the sequence similarity of peptide segments in bacterial and eukaryotic proteomes, most peptide sequences are unique from lengths of about 6–7 amino acids onwards, independent of genome size. Interestingly, this is on the lower length spectrum of linear antibody epitopes, which range from 6 to 25, approximately, suggesting that such short peptides already hold sufficient information for discriminatory binding. In line with this, the immune system uses for self/non-self-discrimination at the cell-surface-bound multihistocompability complexes I and II display peptides of 8–11 and 9–30 amino acids in length, respectively. We noted that APRs, which typically range in length from 5–15 amino acids, follow a similar pattern: APRs above the length of 6-7 amino acids tend to be unique within their proteome, e.g. over 80% of 6-amino acid-long APRs occur only once in the *E. coli* or *S. cerevisiae* genome (Figure 2A) (Ganesan et al., 2015). This is consistent with the previous findings that there is selective pressure to both minimize the aggregation propensity of APRs (Rousseau et al., 2006; Reumers et al., 2009; Ganesan et al., 2016) and avoid identical APRs in repeat-domain proteins (Parrini et al., 2005; Wright et al., 2005). Of course, this relationship is different when one or two mismatches are taken into consideration, but it is at present not possible to predict which mismatches would allow co-aggregation and which ones would not.

A further proof of the selectivity of protein aggregation is that it is possible to use the interaction of APRs with each other for detecting proteins immobilized on a membrane, using the Pep-blot method (Ganesan et al., 2015). Pept-blot is an adapted immunoblot protocol in which the primary antibody is replaced with a biotin-labelled synthetic amyloid peptide. The APR VIIWSLGN from the beta-galactosidase enzyme of *E. coli* is unique within its proteome and there is only one similar APR if we allow 1 mismatch and also one if we allow two mismatches (Figure 2B). Ganesan et al. used the interaction of a biotin-labelled version of the VIIWSLGN peptide to target the beta-galactosidase protein in bacterial lysate immobilized on a membrane and subsequently detected the labelled peptide using streptavidin-conjugated HRP, yielding a single band at the same molecular weight as seen by antibody staining (Figure 2C). The introduction of 2 mutations in the peptide was sufficient to break the interaction. The same approach was used to detect C-reactive protein in human plasma samples and Prostate Specific Antigen in human seminal samples (Ganesan et al., 2015), suggesting amyloid interactions can convey high specificity, at least in these cases.

The combination that amyloid-like aggregation is sequence-specific *and* most APRs are unique within their proteome makes targeted protein aggregation possible. The core of the Pept-in targeted protein aggregation technology invented in our laboratory is supplying short peptides (termed Pept-ins, from *pept*ide *in*terferors) that contain amino acid sequences homologous to the APR of the target protein. Unique APRs can be used as “bar codes” for inducing the specific aggregation of a protein in the proteome by amyloid-like beta-strand self-interaction. In their most basic design, Pept-ins contain a tandem repeat of a 5–7 amino acid long segment of the target APR connected by a linker (Figure 1C). The tandem repeat design of Pept-ins was intended to facilitate the nucleation of the aggregation process and it was inspired by the primary structure of functional amyloids (Shanmugam et al., 2019). Functional amyloids often contain more than one imperfect copies of the same APR, meaning that they contain one or two mismatches between each repeat. For Pept-ins, however we used two perfect copies of the same APR.

Pept-ins are prone to form oligomeric structures although the exact structure of the species that enters the bacteria is not known. To provide colloidal stability to these doubled APR arrangements, each of the APRs in a Pept-in is flanked by charged residues (lysine, arginine, glutamate or aspartate) functioning as aggregation gatekeepers that slow aggregation kinetics (Rousseau et al., 2006; Bednarska et al., 2016; Gallardo et al., 2016). These ensure that while forming oligomers, the particle size remains sufficiently small to form soluble aggregates. The fast aggregation that occurs following Pept-ins treatment suggest that they function as small pre-aggregated seeds for inducing protein aggregation therefore the aggregation of the target protein can skip the rate-limiting nucleation phase and go directly to the fast-growing elongation phase (Figure 1B).

Our lab has generated transgenic *Arabidopsis* and maize plants that, in contrast to a generalized toxicity that might have been expected from aspecific aggregate-interactions, have desirable properties such as increased plant size (Figure 2D) or increased starch production due to the expression of Pept-ins that specifically inactivate BIN2 (an inhibitor of the brassinosteroid growth pathway) and GWD-1 (an inhibitor of the starch biosynthesis pathway), respectively (Betti et al., 2016; Betti et al., 2018).

Subsequently, we designed an anti-tumoral peptide targeting an APR located in the human vascular endothelial growth factor receptor 2 (VEGFR2). This peptide induced the aggregation of VEGFR2, thereby knocking down its function and reducing VEGFR2-dependent growth of tumor allografts of the mouse B16 melanoma line (Figure 2E) (Gallardo et al., 2016). As in the plants, the phenotype in the mammalian cells appeared to agree best with a specific loss-of-function and not a general toxicity: we only observed toxicity of the peptide in cells that depend on VEGFR2 for survival (Figure 2E) but not in cells that do not express VEGFR2 or express VEGFR2 but do not depend on it for their survival (Figure 2F and data not shown).

Most recently, our laboratory has demonstrated that targeting viral proteins using virus-specific amyloids can attenuate the replication of the influenza A and Zika viruses within mammalian cells, by aggregating viral proteins within the mammalian cells (Michiels et al., 2020). Again, the effect was not due to general toxicity. Whereas the antiviral Pept-ins inhibited plaque formation by the influenza A virus, they neither had hemolytic activity (Figure 2G) nor affected the viability of the viral host cells (data not shown).

**FIGURE 3:**
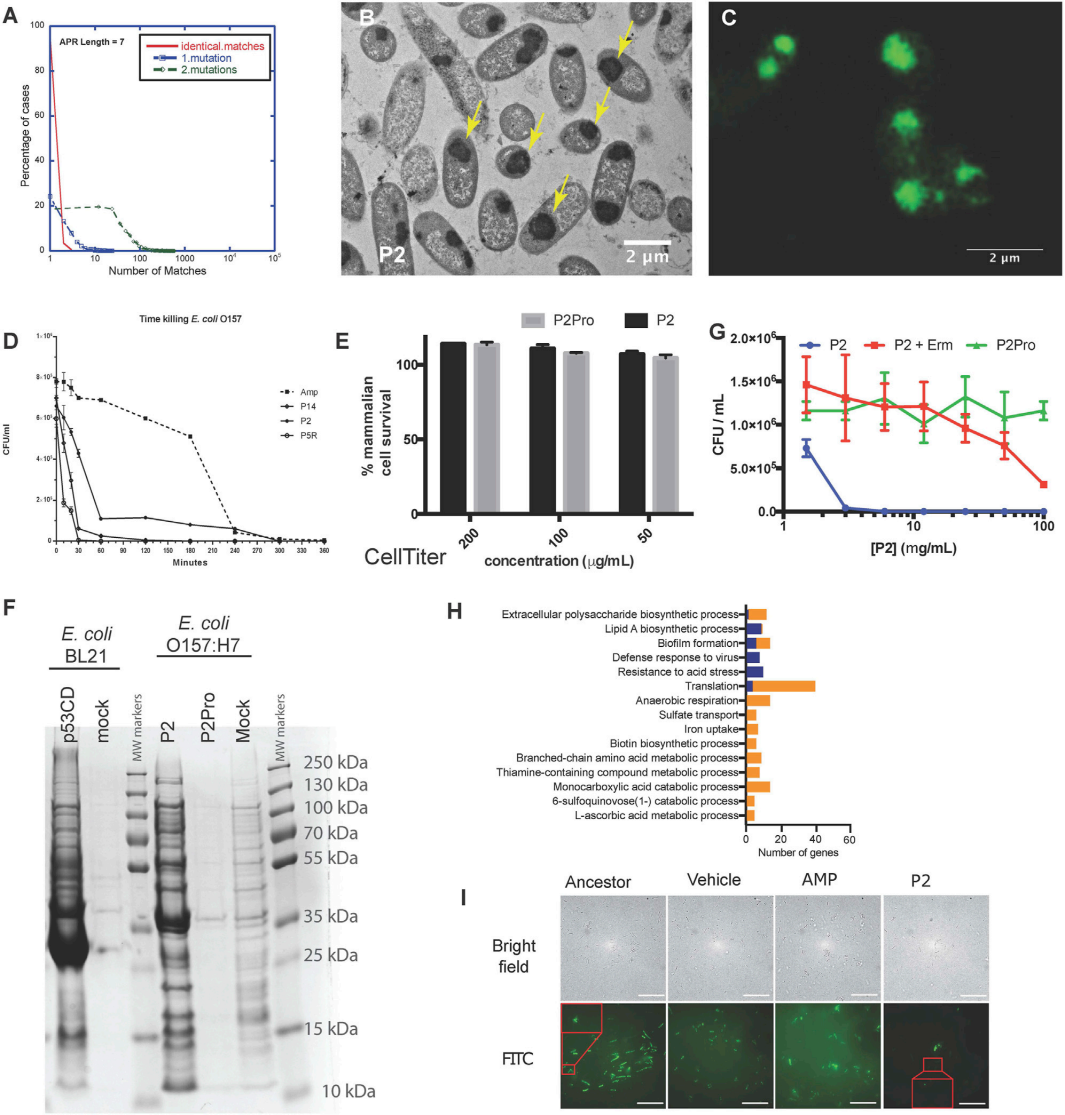
**(A)** A small fraction of APRs are redundant: most APRs of seven amino acids occur in no more than five proteins in the *E. coli* proteome (red curve). The number of homologous APRs in the proteome increases if we allow one mismatch (blue curve) or two mismatches (green). **(B)** Transmission electron microscopy of cross-sections of resin-embedded *E. coli* O157:H7 treated with P2 peptide at MIC concentration for 2 h. The yellow arrows indicate inclusion bodies. **(C)** Wide-field structured illumination microscopy image of *E. coli* O157:H7 treated with peptide P2 and stained with the amyloid-specific dye pFTAA (0.5 µM). **(D)** Time-killing curve of selected peptides (P14, P2, and P5R) and ampicillin (Amp, dashed line) against *E. coli* strain O157:H7 treated at MIC concentration (average ±SD of three replicates). **(E)** Neither P2 (black bars) nor its control variant containing two proline substitutions (P2Pro, grey) is toxic to human HeLa cells as measured using the CellTiter Blue assay. **(F)** Coomassie blue-stained SDS-PAGE of inclusion bodies from *E. coli* BL21-overexpressing the C-terminal domain of human p53 (p53CD, lane 1), mock-transformed (lane 2), and *E. coli* O157:H7 treated with P2 (lane 4), P2Pro (lane 5), or DMSO (lane 6). Molecular weight markers are shown in lanes 3 and 7. **(G)** Growth inhibition of cells treated with P2 with/without erythromycin (Erm, 100 μg/ml, average ±SD of three replicates). (**A–G** Adapted from Khodaparast et al., 2018). **(H)** The number of genes in different gene ontologies expressed differentially in P2-resistant strains compared with ancestors. Blue indicates upregulation, orange indicates downregulation. Apart from the gene ontologies Resistance to acid stress and L-ascorbic acid metabolic process, all other groups had a Bonferroni stepdown *p* value < 0.05. **(I)** Bright field **(upper row)** and wide-field structured illumination microscopy **(lower row)** images of bacteria treated with FITC-labelled P2 peptides for 2 h at 12.5 mg/ml. The Pept-in resistant bacteria (P2) contains much less FITC-P2. Scale bar: 10 µm. (**H,I** Adapted from Wu et al., 2020).

The examples in plants and mammalian cells above showed that synthetic amyloid peptides targeting a specific APR can be used to selectively detect or inactivate proteins containing the same APR by initiating self-assembly. Although most APRs are unique in their proteome, there is a subset of redundant APRs, i.e. that occur in multiple proteins, especially if 1 or 2 mismatches are allowed (Ganesan et al., 2015; Khodaparast et al., 2018) (Figure 3A). We reasoned that targeting these redundant APRs could potentially induce the aggregation of several proteins at the same time, possibly inducing a lethal loss of protein homeostasis. With this in mind, we designed peptides targeting multiple proteins in the Gram-positive *S. aureus* proteome, and identified several that showed strong antibacterial activity, without any major toxicity towards mammalian cells (Bednarska et al., 2016). Similarly, we designed Pept-ins targeting multiple proteins in the proteome of the Gram-negative *E. coli*. We identified several among these that induced the rapid formation of amyloid-like aggregates containing IBs in pathogenic Gram-negative bacteria (Figures 3B,C), apparently ending in the collapse of proteostasis as it caused rapid death of the bacteria, apparently due to loss of proteostasis (Figure 3D) (Khodaparast et al., 2018). Of note, these same peptides induced no aggregation and were not toxic to the mammalian cells tested (Figure 3E).

To better understand the lethal events induced by the peptides, we analyzed IBs isolated from bacteria over-expressing the aggregation-prone C-terminal domain of human p53 (with no major impact on cell viability) and IBs isolated from bacteria treated with peptide P2 (associated to a loss of viability) using SDS-PAGE (Figure 3F), showing that both types of IBs have a complex composition, with major bands corresponding to molecular chaperones. Mass spectrometry-based proteomics comparisons of these same IBs, extracted at a single time point when aggregation was quite advanced, confirmed that the Pept-in induced IBs contained several hundred of bacterial proteins, significantly more than observed in the case of recombinant expression of p53DBD. Of interest, a number of the proteins found in the P2-induced IBs indeed contained similar APRs to the one present in the Pept-in. For example, the Pept-in called P2 that encodes the APR sequence GLGLALV which occurs in the Hcab protein, but also occurs in multiple other proteins if we allow one mismatch. The presence of eight such proteins was confirmed using mass spectrometry in IBs extracted from P2-treated bacteria, suggesting that indeed a multi-targeted induction of aggregation that ends up overwhelming the protein homeostasis could explain the antibacterial effect of P2 (Khodaparast et al., 2018). We have found that there is a common set of over four hundred proteins in the IBs induced by different Pept-ins. A number of these are known to be involved in mediating and controlling IB formation such as molecular chaperones, but others are thought to be proteins that aggregate when the proteostasis machinery is disturbed by the initial aggregation events.

The question remained why IBs induced by Pept-ins disturb bacterial proteostasis so strongly that the bacteria lose viability, whereas other conditions that promote IB formation, such as heterologous expression (Figure 3F), do not appear to be particularly lethal. Part of the answer may be found in the sheer number of proteins found in toxic and non-toxic IBs, which is higher in the toxic case. Importantly, among these there are many more essential gene products in the IBs associated with a loss of viability, suggesting the depletion of critical cellular functions. The surplus proteins belong to various gene ontologies and the deletion of many of them individually is sufficient to impair the viability of the bacteria.

Thus, Pept-ins seem to exert their bactericidal effect by inducing aggregation of a wide range of proteins involved in various essential biological pathways and which ultimately appears to lead to the proteostatic collapse (Khodaparast et al., 2018). Most probably, a similar mechanism (a proteostasis collapse sequestering several essential proteins) was at play during our earlier experiments that demonstrated that aggregation-inducing peptides were effective against *Staphylococcus epidermidis* (Bednarska et al., 2016), although we did not map out the full mechanism of action at that time. The triggers of aggregation at the beginning are probably specific, as evidenced by the presence of the proteins containing homologous APRs in the aggregates. But, as aggregation proceeds and the components of the PN may become less available to chaperone newly made proteins, the aggregation extends to other chaperone-dependent bystander proteins that share no APR similarity with the original trigger.

Various studies have shown that proteins are primarily susceptible for aggregation during translation/folding and proteins that are translated at a higher translation rate tend to aggregate more (Ibstedt et al., 2014; Weids et al., 2016; Hamdan et al., 2017; Liu, 2020). We have also observed that Pept-in-induced aggregation events occur co-translationally. Adding the protein translation inhibitor erythromycin to the Pept-in treatment rendered P2 ineffective (MIC increase from 12.5 to > 100 ug/ml) (Figure 3G) and we observed no Pept-in-induced protein aggregation events in the bacteria, either. Additionally, as mentioned earlier, IBs extracted from Pept-in treated bacteria were strongly enriched in ribosomal proteins, which appears to corroborate that protein aggregation induced by Pept-in treatment occurs co-translationally.

No resistance development to Pept-ins was observed in our studies of wild-type bacteria (Bednarska et al., 2016; Khodaparast et al., 2018) therefore we used a mutator strain to develop strains resistant to Pept-ins. Resistance development was slow and low-grade even in the mutator strain after serial-passaging the bacteria in the presence of sub-MIC concentration of P2 for 27 days (Wu et al., 2020). Comparing the transcriptomic profiles of P2-resistant strains to their ancestors showed that translation was the most affected gene ontology category and translation-related genes were predominantly down-regulated in P2-resistant strains (Figure 3H). This seems to confirm that Pept-ins act co-translationally: reducing translation rates and thereby decreasing the exposure of APRs could rendered bacteria somewhat resistant to Pept-in treatment, but the extent of this potential mechanism is limited since bacteria of course depend on translation for continued survival (Wu et al., 2020). We expected a high translation rate to render bacterial proteostasis more susceptible to perturbation, but confusingly P2 induced a significantly higher amount of aggregation events in the CH184 mutant strain that has a slower translation elongation rate compared to wild-type *E. coli*. This was a surprising result and needs to be further investigated, but seems to confirm that it is the high volume of protein turnover (the high number of polypeptide chains that are in the process of translation at any given time) that makes the proteostasis of bacteria vulnerable and not the high speed of translation itself. Currently, we think that the slower elongation rate in CH184 strain gives P2 a longer time window to act on the unfolded proteins during translation, rendering these proteins more prone to aggregation in the presence of Pept-ins and thus making CH184 more susceptible towards Pept-ins (Wu et al., 2020).

Since Pept-ins seem to disrupt bacterial homeostasis via inducing widespread bacterial protein aggregation, modification of the target proteins seems an obvious way to increase survival during Pept-in treatment. However, this resistance mechanism was not observed in the resistant strains, indicating a) the clear benefits of designing antibiotics targeting a large number of targets and b) the difficulty of changing the targeted regions (the APRs that form part of the hydrophobic core of the protein) because this usually requires multiple mutations (Langenberg et al., 2020). Phenotypic, lipidomic, transcriptomic, as well as genotypic changes of laboratory-derived Pept-in-resistant *E. coli* mutator cells revealed that preventing uptake was the main resistance mechanism to Pept-ins (Wu et al., 2020) (Figure 3I).

## Conclusion

Since the evolution of resistance to antibiotics seems inescapable, we need to find antimicrobials that can be developed at a high rate and for which it takes a longer time for resistance to occur (McClure and Day, 2014). Pept-ins score high on both of these scales. Also, Pept-ins have a novel mode of action and can target intracellular proteins, even in Gram-negative strains where this is notoriously difficult. Upon intravenous injection in preclinical models, Pept-ins were able to reach an effective concentration *in vivo* at the infection site to eliminate pathogens (Bednarska et al., 2013; Khodaparast et al., 2018), suggesting that they may exhibit a more beneficial biodistribution than might be expected from their peptidic nature. The resistance frequency observed with Pept-ins thus far appears to be low, probably due to their multiple targets and the fact that changing the targeted region in each target requires multiple mutations.

Because of all these properties, and their designability that allows tuning of the degree of specificity and cross-reactivity, Pept-ins represent a promising novel class of antibiotics and are excellent candidates for evolving them into a drug development platform for the rapid design and development of new antimicrobial peptides in response to the emergence of pathogens. However, Pept-ins may face similar challenges as other peptide drugs, most notably fast metabolism and rapid elimination (Craik et al., 2013), which may limit their *in vivo* effectiveness and the possibility of being orally administrated as a systemic medication.

As we have seen above, the major steps towards bacterial death during Pept-ins treatment are the aggregation of a large number of proteins and the formation of IBs. This mechanism of action is somewhat surprising because bacteria have very well developed stress-responses to deal with protein aggregation (increasing both the levels of chaperones and the disaggregation machinery) (Schramm et al., 2019) and IBs are generally regarded as not toxic. The question remains: how does the aggregation of a large number of proteins become lethal to the bacteria?

One possibility is that the widespread protein aggregation induced by the Pept-in removes some protein(s) from the cytosol of the bacteria that is/are essential for the survival of the organism. This is certainly possible since we could identify essential proteins trapped in the IBs whose individual deletion impairs the viability of the bacteria. Another possibility is that the widespread protein aggregation caused by Pept-ins ties down cellular resources in general, as put forth by the chaperone competition hypothesis (Sinnige et al., 2020). According to this hypothesis, when something shifts the balance of the PN towards aggregation, the competition between misfolded proteins and endogenous clients for the limited pool of available chaperones will have consequences on protein functionality in general. Although stress responses can increase the pool of available PN components many-fold, there is evidence that the cellular resources can wear too thin to maintain proteostasis. For example, very high-level expression of so-called gratuitous gene products (proteins that are not toxic but have no function for the cell) leads to the destruction of the ribosomes and loss of translation capacity (Dong et al., 1995). Also, it is known that in case the expression of a recombinant protein induces IB formation, one of the troubleshooting steps to try is co-express chaperones because this can help to keep the recombinant protein in solution (Rosano and Ceccarelli, 2014), indicating again that the expression of one single protein in large quantities can exhaust the pool of available chaperones. Also, our earlier results indicated that chaperone dependency of bacterial proteins correlated most strongly with protein abundance (Ramakrishnan et al., 2019) which meshes very well with our experience in the design of Pept-ins, namely that targeting abundant proteins usually yields Pept-ins that are more toxic to the bacteria.

Chaperone-client interactions are normally transient in nature and a limited pool of chaperones can serve a large pool of client proteins. During large-scale protein aggregation, the aggregated proteins sequester chaperones and the transient chaperone-client interactions become permanent ones, as evidenced by the presence of chaperones within the aggregates. The loss of chaperone function upon protein aggregation then leads to the misfolding of other proteins exacerbating general cellular toxicity. An analogous process was uncovered in worms where the decline of the proteostasis starts already in early adulthood but it does not lead to problems for the organism until only later on when the ability of the PN to respond declines (Ben-Zvi et al., 2009). Moreover, the aggregates also interfere with protein degradation by the proteasome and autophagy systems. Aggregates that are originally the symptom of a proteostasis imbalance then become the cause of it because the aggregates tie up PN components, and interfere not only with the degradation of other substrates and but with the folding of other proteins, as well, by sequestering chaperones (Hipp et al., 2014)—setting in motion a vicious cycle that ultimately triggers proteostasis collapse (Sinnige et al., 2020).

As discussed earlier, once amyloid fibers are formed, they can template the addition of further protein monomers (Soto and Pritzkow, 2018; Lutter et al., 2019). This can lead to the gain of toxic function of protein aggregates: other proteins can engage in beta-strand interactions with the exposed active elongation sites, leading to their deposition in the aggregates. This toxic function may be completely unrelated to the original function of the aggregated protein (Balchin et al., 2016).

Bacteria can usually deal with IBs very well: although there is an inverse relationship between aggregate content of bacteria and their viability (Maisonneuve et al., 2008), aggregates usually remain in the old pole cell, leaving the young daughter cells fit and free of aggregates (Sabate et al., 2010; Fay and Glickman, 2014; Vaubourgeix et al., 2015). There is data showing that bacteria causing chronic infections can survive for prolonged periods of host-imposed stresses in combination with antibiotic treatment by using the mentioned asymmetrical distribution of aggregates, giving the daughter cells inheriting less of the damaged proteins a growth advantage (Vaubourgeix et al., 2015). Why are Pept-in-induced IBs lethal, then?

As mentioned, the elongation phase of protein aggregation can proceed very quickly once enough seeds are available. Pept-ins serve as seeds for aggregation and the speed of aggregation may be a deciding factor. Bactericidal Pept-ins seem to initiate very fast and widespread protein aggregation that ripples through the proteome quickly and causes the collapse of the proteostasis before bacteria have time to jettison aggregated proteins by dividing and producing new, aggregate-free daughter cells. Moreover, we observe inclusion bodies in both cell poles in many cells, suggesting that symmetric segregation of proteome damage to one daughter cell may not be possible, and finally, the total number of proteins in the IBs induced by Pept-ins is very high, suggesting a widespread loss of function throughout the proteome.

In summary, what Pept-ins taught us about bacterial proteostasis is, that, despite all the redundancy built in the PN of bacteria, and its great capacity for expansion, it is possible to overwhelm bacterial proteostasis and induce a proteostasis collapse that leads to the death of bacteria, if 1) the number of different proteins that aggregate is high enough and 2) the aggregation happens fast enough so that the bacteria do not have time to catch up with the backlog of aggregated proteins by slowing down the translation rate or get rid of the mass of aggregated proteins by asymmetrical division.

## Abbreviations

PN, proteostasis network; IB, inclusion body; APR, aggregation-prone region; Pept-in, peptide interferor; MIC, minimal inhibitory concentration
